# Knock‐out of MicroRNA 145 impairs cardiac fibroblast function and wound healing post‐myocardial infarction

**DOI:** 10.1111/jcmm.15597

**Published:** 2020-07-06

**Authors:** Hui‐Fang Song, Sheng He, Shu‐Hong Li, Jun Wu, Wenjuan Yin, Zhengbo Shao, Guo‐qing Du, Jie Wu, Jiao Li, Richard D. Weisel, Subodh Verma, Jun Xie, Ren‐Ke Li

**Affiliations:** ^1^ Department of Anatomy Shanxi Medical University Taiyuan China; ^2^ Department of Biochemistry and Molecular Biology Shanxi Key Laboratory of Birth Defect and Cell Regeneration Shanxi Medical University Taiyuan China; ^3^ Toronto General Research Institute University Health Network Toronto ON Canada; ^4^ Division of Cardiac Surgery Department of Surgery University of Toronto Toronto ON Canada; ^5^ Division of Cardiac Surgery Li Ka Shing Knowledge Institute of St Michael's Hospital Department of Surgery University of Toronto Toronto ON Canada

**Keywords:** miR‐145, myocardial infarction, myofibroblast, transdifferentiation, wound healing

## Abstract

Prevention of infarct scar thinning and dilatation and stimulation of scar contracture can prevent progressive heart failure. Since microRNA 145 (miR‐145) plays an important role in cardiac fibroblast response to wound healing and cardiac repair after an myocardial infarction (MI), using a miR‐145 knock‐out (KO) mouse model, we evaluated contribution of down‐regulation of miR‐145 to cardiac fibroblast and myofibroblast function during adverse cardiac remodelling. Cardiac function decreased more and the infarct size was larger in miR‐145 KO than that in WT mice after MI and this phenomenon was accompanied by a decrease in cardiac fibroblast‐to‐myofibroblast differentiation. Quantification of collagen I and α‐SMA protein levels as well as wound contraction revealed that transdifferentiation of cardiac fibroblasts into myofibroblasts was lower in KO than WT mice. In vitro restoration of miR‐145 induced more differentiation of fibroblasts to myofibroblasts and this effect involved the target genes Klf4 and myocardin. MiR‐145 contributes to infarct scar contraction in the heart and the absence of miR‐145 contributes to dysfunction of cardiac fibroblast, resulting in greater infarct thinning and dilatation. Augmentation of miR‐145 could be an attractive target to prevent adverse cardiac remodelling after MI by enhancing the phenotypic switch of cardiac fibroblasts to myofibroblasts.

## 1 INTRODUCTION

Myocardial infarction (MI) remains the leading cause of death worldwide. Adverse cardiac remodelling after MI resulting from infarct thinning and expansion contributes to ventricular dilatation and heart failure which can result in disability and death.^1^, ^2^, ^3^ The mechanisms underlying cardiac remodelling are complex, but the contractile insufficiency and ventricular dilation remain the most important contributors to adverse outcomes.^4^

Augmentation of cardiac contractile function may improve outcomes, but clinical translation of this strategy has not been successful after an MI.^5^, ^6^ However, a number of new technologies continue to be evaluated, such as improving calcium handling.^1^ Other therapeutic interventions have also been evaluated, including stem cell therapy to improve wound healing and to prevent ventricular remodelling,^5^, ^7^, ^8^, ^9^, ^10^ and altering molecular pathways to reduce the extent of cardiomyocyte necrosis.^11^, ^12^ Although several discoveries are promising, none have become standards of clinical care and many patients continue to suffer from congestive heart failure after MI despite medical therapy. Novel strategies to prevent cardiac dilatation and dysfunction are required to complement existing treatments for reverse the trend of increasing morbidity and mortality from heart failure.

Cardiomyocytes loss as a result of ischaemia initiates a series of responses to repair the infarct and heal the scar. Cardiac fibroblasts play an important role in wound healing after MI.^13^, ^14^ The fibroblasts are a dynamic cell type and can differentiate into myofibroblasts in response to injury, to mediate wound healing and tissue repair.^15^, ^16^, ^17^ As myofibroblasts, they synthesize intracellular contractile proteins, such as α‐SMA and they generate increased levels of extracellular proteins, such as collagen. These activities contribute to reparative fibrosis and the formation of a stable scar which prevents ventricular rupture and may limit infarct expansion and ventricular dilatation.^15^ We believe that promoting differentiation of fibroblasts to myofibroblasts might be an effective strategy to stimulate infarct scar contracture, preserve ventricular morphology after injury and prevent progressive heart failure.

MicroRNA 145 (miR‐145) regulates the proliferation and differentiation of a variety of cells,^18^, ^19^ including endothelial and smooth muscle cells (SMCs) in the vascular system.^20^, ^21^, ^22^, ^23^ In vascular SMCs, deletion of MiR‐143/145 resulted in severe reduction in the number of contractile SMCs.^24^ In the current study, using a miR‐145 knock‐out (KO) mouse model, we investigated the effects of miR‐145 deletion on myofibroblast formation during adverse cardiac remodelling after an MI and evaluated potential mechanisms.

## 2 MATERIALS AND METHODS

### 2.1 Experimental animals

All animal procedures were approved by the University Health Network Animal Care Committee. All experiments were carried out according to the Guide for the Care and Use of Laboratory Animals (NIH, 8th Edition, 2011). MiR‐145 KO mice were generated as previously described^25^ and maintained in a C57BL/6 background. Briefly, miR‐145 was deleted in mice, by introducing loxP sites for Cre‐mediated recombination at the regions flanking the pre‐miR coding regions for homologous recombination. The targeting strategy deleted the 70‐bp pre‐miR stem‐loop sequence of miR‐145, or a genomic region encompassing miR‐145, and replaced it with a neomycin resistance cassette flanked by loxP sites. The neomyocin resistance cassette was removed by breeding these mice with mice expressing a ubiquitously‐expressed CAG‐Cre transgene. Breeding the offspring of these crosses generated homozygous mutants with a ubiquitous knock‐out of miR‐145. WT littermates were also from a C57BL/6 background.

The left coronary artery of mice was permanently ligated to induce an anterior MI. During this procedure, mice were incubated and ventilated with 2% isoflurane (Pharmaceutical Partners of Canada Inc). The infarct size in each mouse was estimated immediately after coronary artery ligation by visual identification of the tissue discoloration (loss of the normal pink colour and accumulation of a blue colour). Mice with infarct sizes between 30% and 35% of the left ventricular free wall were used in the following experiments. WT littermates receiving the same procedure, though without ligation of the left coronary artery, were used as sham control.

Cardiac function was measured with echocardiography before and 7, 14, 21 and 28 days after MI and with a pressure‐volume catheter 28 days after MI. The following parameters were calculated by echocardiography: Left ventricular internal systolic dimension (LVIDs), left ventricular internal diastolic dimension (LVIDd) and percentage of fractional shortening (fractional shortening %) = (LVIDd‐LVIDs)/LVIDd*100. Pressure‐volume analysis was used to determine ejection fraction, dP/dt, tau and left ventricular (LV) volumes. At the end of the study (28 days after MI), the hearts were arrested and fixed at physiologic pressures. The LV was serially sectioned into five 1 mm rings along the longitudinal axis, representing cross‐sections of that region of the heart. The scar area was calculated by computerized planimetry (Image‐Pro Plus, Media Cybernetics) of digital images, consisting of three Masson's trichrome‐stained serial LV sections from the apex. The scar area of each heart section was measured by scar tissue length (epicardial scar + endocardial scar lengths)/2 × 1 mm of section thickness. Total scar area was calculated as the sum of the scar area of each left ventricle cross‐section ring, divided by the sum of the left ventricular free wall area (epicardial + endocardial lengths)/2 × 1 mm thickness of each ring section. Scar thickness was measured from the second ring of the serial LV sections from the apex, with an average of three measurements, one at the middle, and two at the edges of the scar region within that section. Remote myocardial wall thickness was measured from the interventricular septum of the third ring in the LV serial sections from the apex, with an average of three measurements, one at the middle, and two at the edges of the remote region within that section. The Masson's trichrome staining was conducted to show the fibrotic tissue and the scar area in both WT and miR‐145 KO mice. The heart tissues were collected at 3,7,14 and 28 days after MI for miRNA detection. For immunostaining, hearts were collected and fixed at 7 days after MI at physiological ventricular pressures to preserve ventricular geometry.

### 2.2 Total RNA extraction and real‐time qPCR

Total RNA was extracted from mouse heart tissue or cells with Trizol reagent (Sigma‐Aldrich, T9424) according to the manufacturer's instructions. Expression of miR‐145 was evaluated by the TaqMan microRNA Assay kit (Thermo Fisher scientific, 4366596 for Taqman microRNA reverse transcription and 4324018 for quantitative PCR). U6 snRNA was used as a control. Total RNA was converted to cDNA and the expression of myocardin was detected by real‐time qPCR with the fast SYBR Green master mix kit. GAPDH was served as the loading control. Primer pairs specific for mouse myocardin A (sense primer: 5′‐CTTCTCTCCCCCAGCTTCCA‐3′; antisense primer: 5′‐CTTGGGCTTTTGGGACAAGG‐3′) and GAPDH (sense primer: 5′‐AGAACATCATCCCTGCATCC‐3′; antisense primer: 5′‐CACATTGGGGGTAGGAACAC‐3′). The primers of miR‐145 and U6 were purchased from Thermo Fisher scientific. Relative expression was calculated using the 2^‐ΔΔCt^ method.

### 2.3 MiR‐145 mimic transfection

Chemically modified sense RNA (miR‐145 mimic) was synthesized by Qiagen and the sequence is 5′‐GUCCAGUUUUCCCAGGAAUCCCU‐3′. Transfection with the miR‐145 mimic (5 nmol/L) was performed using HiPerFect transfection reagent (Qiagen) following the manufacturer's instructions. The cells were harvested 48 hours after transfection and used for the following experiments.

### 2.4 Protein isolation and western blotting

Total protein was extracted from cells by lysis buffer (50 mmol/L Tris, 1% NP‐40, 150 mmol/L NaCl, 1 mmol/L EDTA, 1 mmol/L β‐glycerolphosphate, pH 7.4) with protease and phosphatase inhibitors. Equal amounts of protein were loaded onto SDS‐polyacrylamide gel and transferred to PVDF membranes. After blocking, membranes were incubated with primary antibodies overnight at 4°C, followed by incubation with HRP‐conjugated secondary antibodies. An enhanced ECL Western blotting detection reagent was used to detect signals. Antibodies used including: collagen I (Cat#: AB21286, Abcam); α‐SMA (Cat#: A2547, Sigma); atrial natriuretic peptide (ANP, Cat#: AB5490‐I) and Klf4 (Cat#: AB72543, Abcam). The density of bands was obtained by ImageJ software (NIH), and protein expression level was calculated using the ratio to the loading control (GAPDH, Cat#: MAB374).

### 2.5 Collagen gel contraction assay

Collagen type I from rat tail was obtained from Corning (Cat#: 354249). Cardiac fibroblasts, which were isolated from WT or miR‐145 KO mice, transfected with 5 nmol/L miR‐145 mimic or scrambled miRNA were mixed with the neutralized collagen solution. The mixture with cardiac fibroblasts or no cells (blank) were placed into a 24‐well plate and allowed to form gels at room temperature in the hood. Then culture medium was added, and the plate was placed into the incubator for 24 hours. Gel size was measured and the percentage of gel shrinkage was calculated.

### 2.6 Cell migration assay

Cardiac fibroblasts from different groups grown to confluence in 35 mm plates were scratched with a sterile pipette tip, washed twice with PBS and incubated in serum‐free medium at 37°C for 24 hours. Pictures were taken at 40× magnification, and the migration rate was calculated using ImageJ software (NIH). Then the cells were fixed and stained, and the percentage of polarized and α‐SMA^+^ polarized cells was calculated.

### 2.7 Immunofluorescence staining

Paraffin‐embedded mouse heart slices or cardiac fibroblasts were fixed with 2% paraformaldehyde in PBS for 20 minutes at room temperature. The fixed cells or slices were permeabilizated with 0.2% Triton X‐100 in PBS and blocked for 1 hour in PBS containing 10% bovine serum albumin. They were then incubated with primary antibody α‐SMA (Cat#: A2547, 1:800, Sigma) overnight at 4°C. Incubation with Alexa546 donkey anti‐mouse (Cat#: A10036, 1:400, Invitrogen) secondary antibody was carried out at room temperature for 1 hour. Fluorescein‐conjugated phalloidin (F‐actin; Cat#: F432, Invitrogen) was used to stain the cytoskeleton structure, and the nuclei were identified with DAPI (Sigma).

### 2.8 Statistical analyses

All values were expressed as mean ± SD. Analyses were performed with SPSS software. Student's *t* test was used for 2‐group comparisons. Comparisons among three or more groups were analysed using one‐way analysis of variance (ANOVA) or two‐way ANOVA with repeated measures over time, followed by Tukey *post hoc* tests. Differences were considered statistically significant at *P* < .05.

## 3 RESULTS

### 3.1 Temporal change in miR‐145 expression after MI in WT mice

**FIGURE 1:**
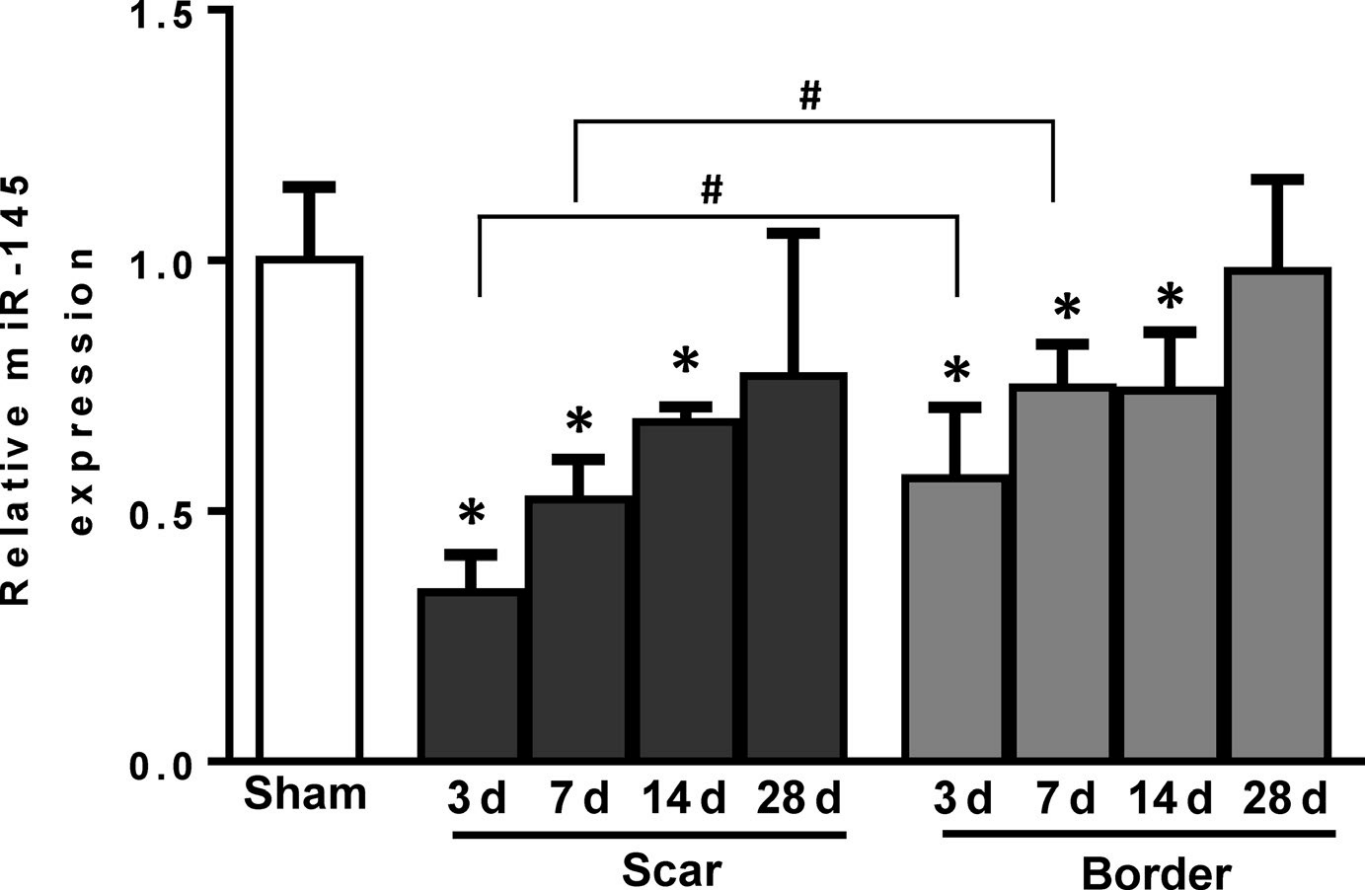
Temporal change in miR‐145 expression after MI in WT mice. miR‐145 expression in both the scar and border region decreased 3 d post‐myocardial infarction (MI), then increased gradually from Days 7‐14 and was restored by 28 d post‐MI in wild type (WT) mice. *P < .05 vs sham group, # *P* < .05 vs border zone at corresponding time point, n = 6‐12/group

To investigate role of miR‐145 in cardiac remodelling after MI, miR‐145 expression in both the scar and border regions of WT mice was measured and compared to the expression in sham‐operated animals (Figure 1). MiR‐145 levels in both the scar and border regions significantly lower at 3 days post‐MI and then increased gradually from days 7‐28 post‐MI in WT mice. In addition, miR‐145 expression in the scar area, at 3 and 7 days post‐MI, was significantly lower, in comparison to the border area at the corresponding time point. The temporal change and the complete restoration of miR‐145 expression by 28 days post‐MI suggest that miR‐145 may take part in the post‐infarction remodelling. We postulated that in accordance to the course of MI, miR‐145 was down‐regulated as the result of massive cell death, particularly from fibroblasts, during the initial course of MI. As MI progresses and fibroblasts started to proliferate to compensate for the lost parenchymal cells, those fibroblasts make miR‐145 as a required factor in mediating fibroblast‐to‐myofibroblast transdifferentiation for subsequent scar contraction.

### 3.2 Cardiac function decreased more in miR‐145 KO than WT mice

**FIGURE 2:**
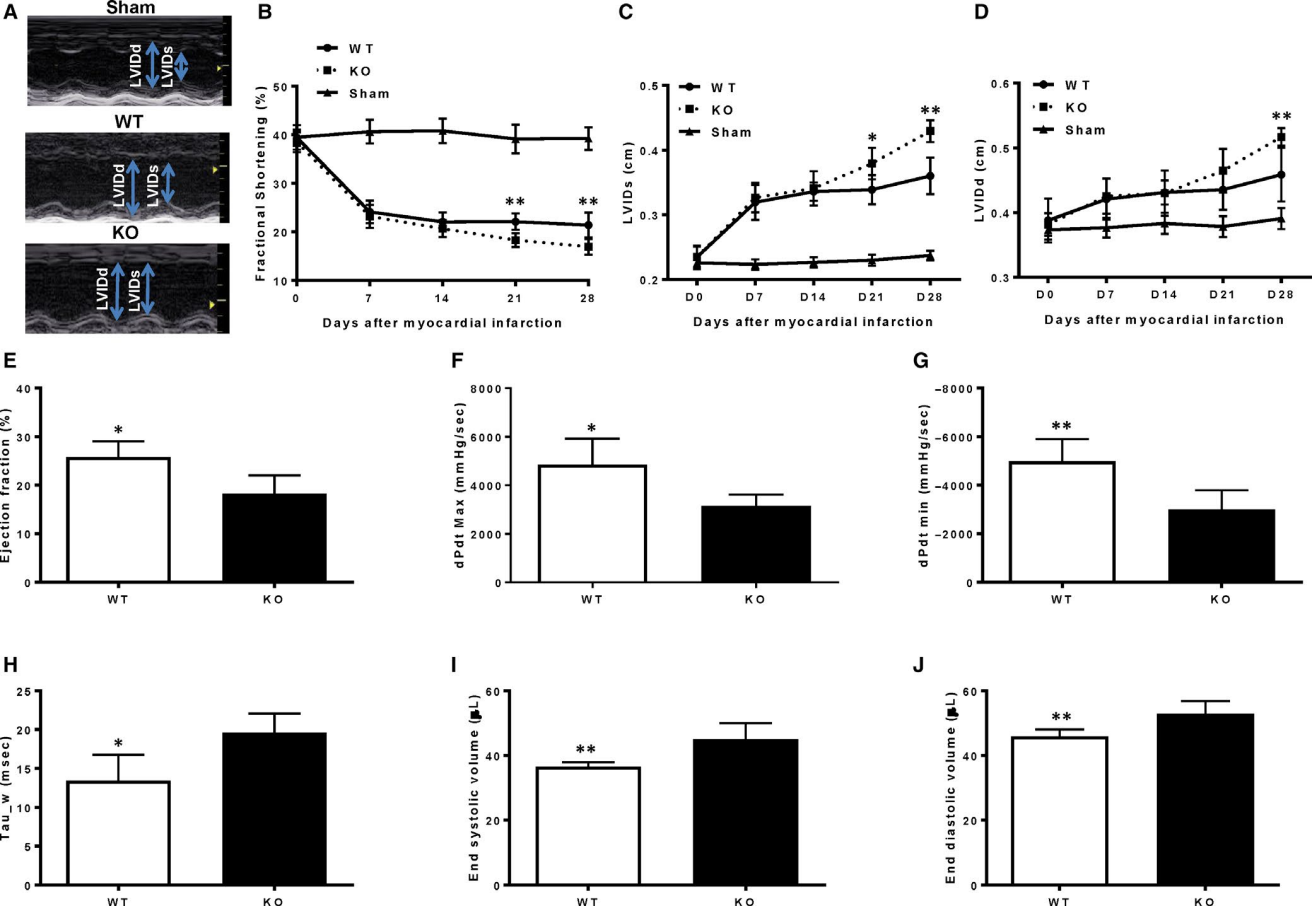
Cardiac function decreased more in miR‐145 KO than WT mice assessed by echocardiography and pressure‐volume loop analysis 28 d after MI. (A) Representative M‐mode echocardiographic images taken 28 d post‐myocardial infarction (MI) in wild type (WT) without MI (sham), WT, and miR‐145 knock‐out (KO) mice. Evaluated by echocardiography, the fractional shortening (B) was significantly lower at 21 and 28 d post‐MI in KO than WT mice. The left ventricle internal systolic dimension (LVIDs) was significantly higher in KO than WT mice at 21 and 28 d post‐MI (C) and the left ventricle internal diastolic dimension (LVIDd) was significantly higher in KO than WT mice 28 d post‐MI (D). Evaluated by pressure‐volume loop analysis, the ejection fraction (E), dP/dt max (F) and dP/dt min (G) were significantly lower in KO than WT mice 28 d post‐MI. The Tau_w (H), end systolic volume (I) and end diastolic volume (J) were significantly higher in KO than WT mice 28 d post‐MI. *P < .05, **P < .01, n = 6/group

To evaluate effect of miR‐145 on cardiac remodelling and function, we compared miR‐145 KO mice to WT at different time points post‐MI. WT mice without MI were treated as sham controls. Cardiac function was determined by echocardiography (Figure 2A‐D) and pressure‐volume loop analysis (Figure 2E‐J). M‐mode echocardiographic images were captured 28 days post‐MI (representative images are displayed in Figure 2A). All echocardiographic parameters were similar between KO and WT mice before MI (Day 0) and changed to a similar degree at 7 days following MI. However, the decline in fractional shortening was greater in miR‐145 KO than in WT mice and was significantly lower at 21 and 28 days post‐MI in KO than WT mice (Figure 2B). MI resulted in progressive left ventricular dilation and the enlargement is greater in KO mice. The LVIDs was significantly larger in KO than WT mice at 21 and 28 days post‐MI (Figure 2C) and the LVIDd was significantly higher in KO than WT mice at 28 days post‐MI (Figure 2D). At 28 days after MI, the invasive pressure‐volume loop analysis was performed. The results showed that systolic function indices including ejection fraction (Figure 2E) and dP/dt max (Figure 2F) were significantly lower in KO than WT mice at 28 days post‐MI. The load‐dependent index dP/dt min (Figure 2G) was significantly lower and load‐independent index Tau_w of diastolic function (Figure 2H) was significantly higher in KO than WT mice at 28 days post‐MI. The end systolic volume (Figure 2I) and end diastolic volume (Figure 2J), indices of left ventricle dilatation, were significantly higher in KO than WT mice at 28 days post‐MI. These results suggested that both systolic and diastolic functions were worse, and LV dilation was more severe in miR‐145 KO mice.

### 3.3 Increased infarct scar and decreased myofibroblast formation in miR‐145 KO mice

**FIGURE 3:**
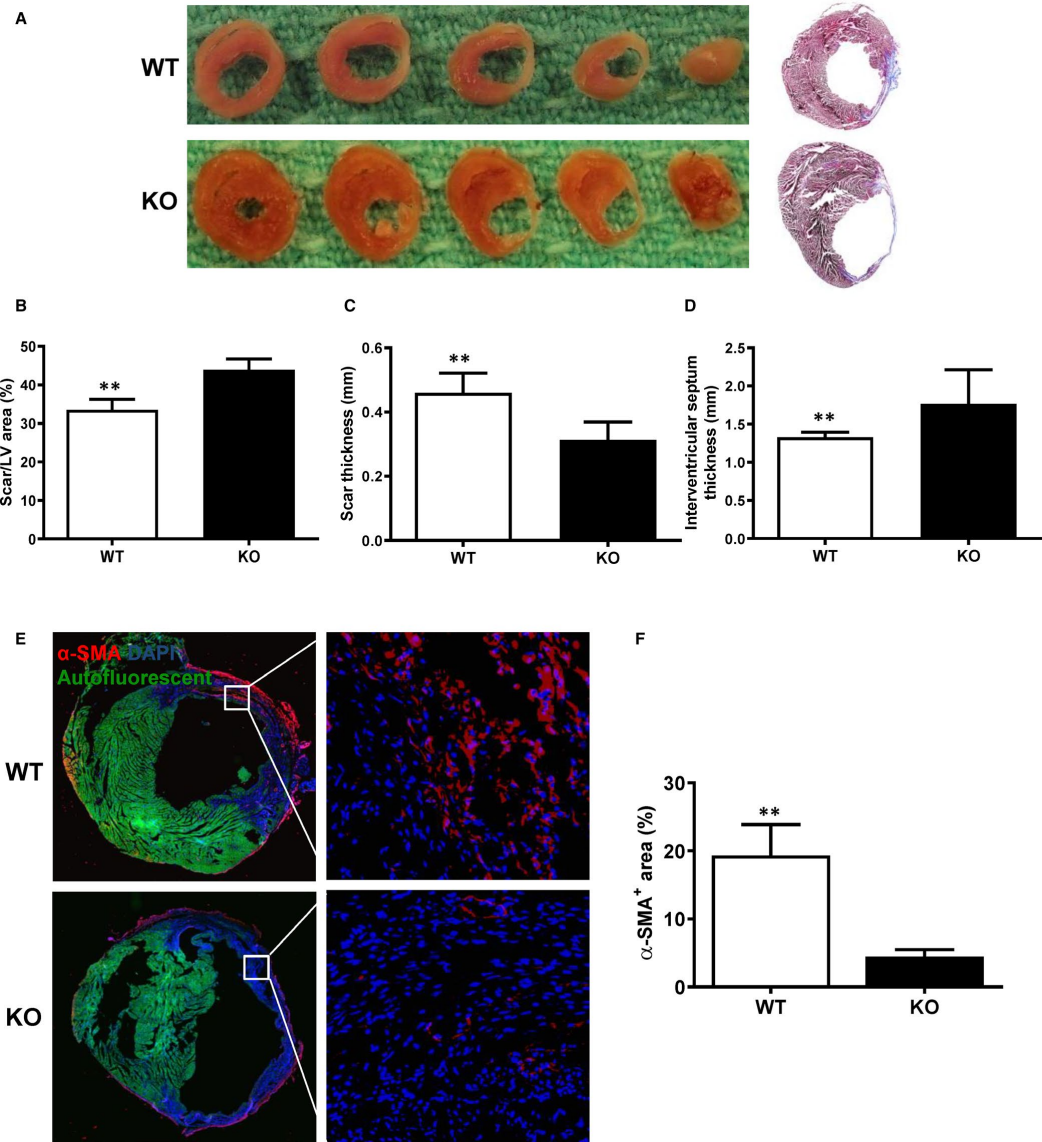
Larger scar size was associated with fewer α‐SMA‐positive cells in miR‐145 KO after MI. Myocardial infarction (MI) was induced in wild type (WT) and miR‐145 knock‐out (KO) mice. (A) Representative images of whole sectioned hearts and mid‐papillary transverse sections stained with Masson's trichrome at 28 d post‐MI. (B) The scar size area was larger in KO than WT mice 28 d post‐MI. (C) The scar thickness was thinner in KO than WT mice 28 d post‐MI. (D) The remote myocardial wall thickness (interventricular septum thickness) of the miR‐145 KO was significantly larger compared to that in WT mice at 28 d post‐MI. (E) Representative micrographs of immunofluorescent staining for α‐smooth muscle actin (α‐SMA, red) to determine the transdifferentiation of cardiac fibroblasts into myofibroblasts. Nuclei stained blue with DAPI. (F) Quantification of the staining showed that the α‐SMA‐positive area in the whole heart at Day 7 post‐MI was significantly lower in KO than WT mice **P < .01, n = 5/group for B, Cand D, n = 3/group for F

After functional analysis, the hearts of both KO and WT mice were fixed at physiological pressures and images of whole sectioned hearts were taken at the mid‐papillary transverse sections and stained with Masson's trichrome. The results showed that the scar area was larger and the scar thickness was thinner in KO than WT mice at 28 days post‐MI (Figure 3A‐C). On the other hand, myocardial wall thickness of non‐infarcted region of the miR‐145 KO mice was significantly larger compared to that in WT mice at 28 days post‐MI, suggesting possible myocardial hypertrophy in the miR‐145 KO mice (Figure 3D). When evaluated by Western blots, levels of ANP, an indicator of cardiac hypertrophy, were significantly higher in miR‐145 KO compared to WT mice, especially in the remote region (Figure S1). These results further supported our observations that increased scar thinning and dilatation were associated with the thickening of the LV remote region, owing to compensatory cardiac hypertrophy. To evaluate correlation between increased scar area and miR‐145‐mediated myofibroblast differentiation, hearts of WT and KO mice at Day 7 after MI were used for immunofluorescent staining for α‐SMA, to determine the extent of transdifferentiation of cardiac fibroblasts into myofibroblasts (Figure 3E). Quantification of α‐SMA‐positive area (which represents myofibroblasts) in the whole heart was lower in KO compared with WT mice, indicating that transdifferentiation of cardiac fibroblasts into myofibroblasts after MI was less in miR‐145 KO than in WT mice (Figure 3F). The decreased myofibroblast differentiation and the increased remote myocardial wall thickness in KO mice may impair infarct scar contraction and promote adverse cardiac remodelling after MI.

### 3.4 Knock‐out of miR‐145 impairs the functional conversion of cardiac fibroblasts to myofibroblasts

**FIGURE 4:**
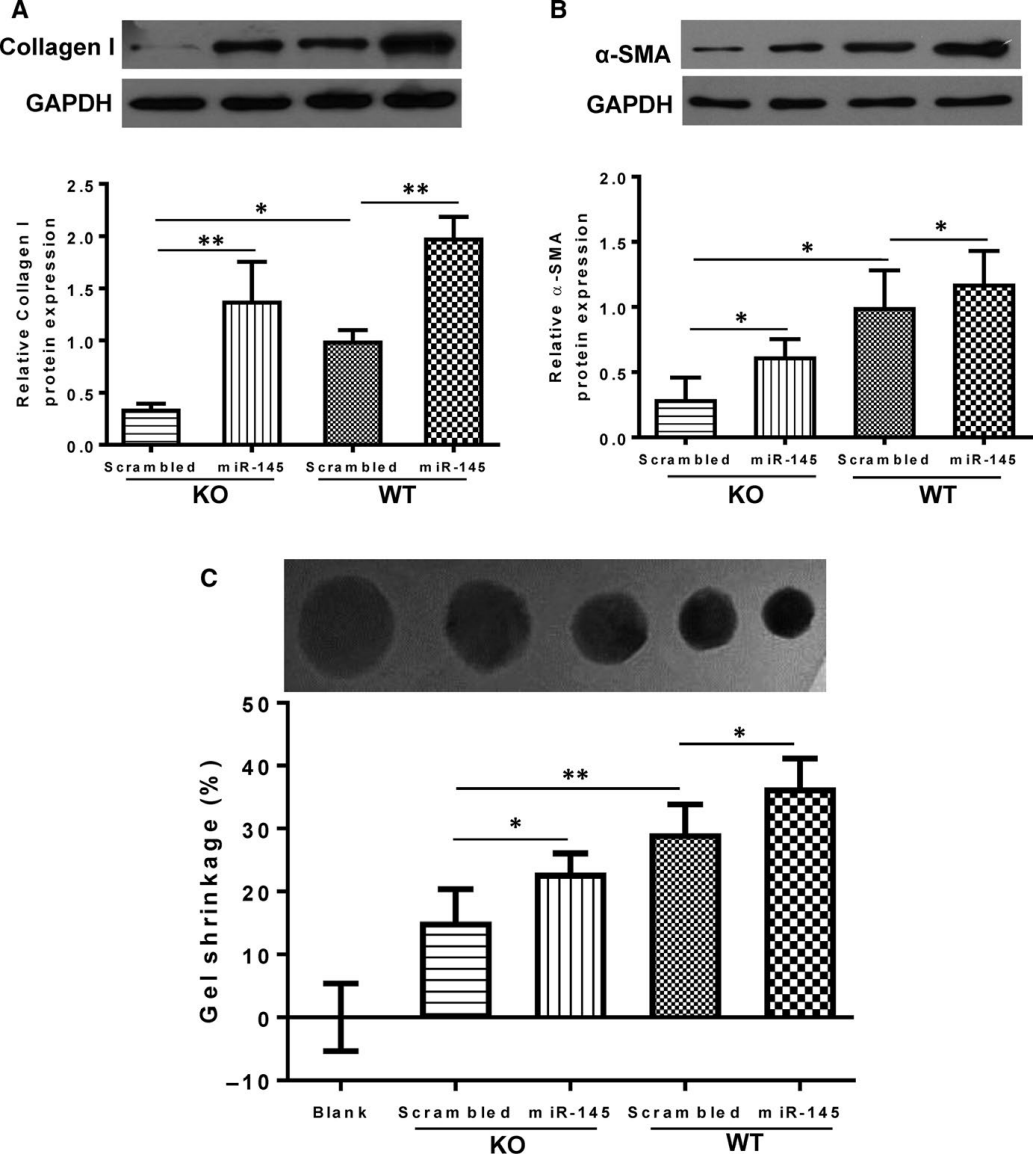
Effects of miR‐145 on the transdifferentiation of cardiac fibroblasts into myofibroblasts. The protein expression of collagen I (A) and α‐SMA (B) in cardiac fibroblasts of miR‐145 knock‐out (KO) mice treated with a scrambled miRNA was significantly lower than in wild type (WT) mice treated with a scrambled miRNA. Treatment with a miR‐145 mimic partially restored the expression of collagen I and α‐SMA in KO cardiac fibroblasts. Cardiac fibroblast‐mediated collagen gel contraction (detected by a collagen gel contraction assay) in KO treated with a scrambled miRNA was significantly less than in WT mice treated with a scrambled miRNA. Treatment with a miR‐145 mimic partially restored the ability of KO cardiac fibroblasts to contract the collagen gel (C). *P < .05, **P < .01, n = 3/group for A and B, n = 4/group for C

To further confirm our in vivo finding, we harvested cardiac fibroblasts from WT and miR‐145 KO mice and treated them with a miR‐145 mimic or a scrambled miRNA as control. The protein expression of mature collagen I and α‐SMA, which are expressed after the conversion of fibroblasts to myofibroblasts, was significantly lower in KO than in WT cardiac fibroblasts, indicating the spontaneous myofibroblast transdifferentiation in KO is lower than in WT mice (Figure 4A‐B). Treatment with a miR‐145 mimic promoted the conversion of fibroblasts to myofibroblasts by increasing the expression of collagen I and α‐SMA in both WT and KO fibroblasts. The miR‐145 mimic treatment partially restored the expression of collagen I and α‐SMA in cardiac fibroblasts of KO mice, further confirming the involvement of miR‐145 in the process of transdifferentiation of cardiac fibroblasts into myofibroblasts (Figure 4A‐B).

**FIGURE 5:**
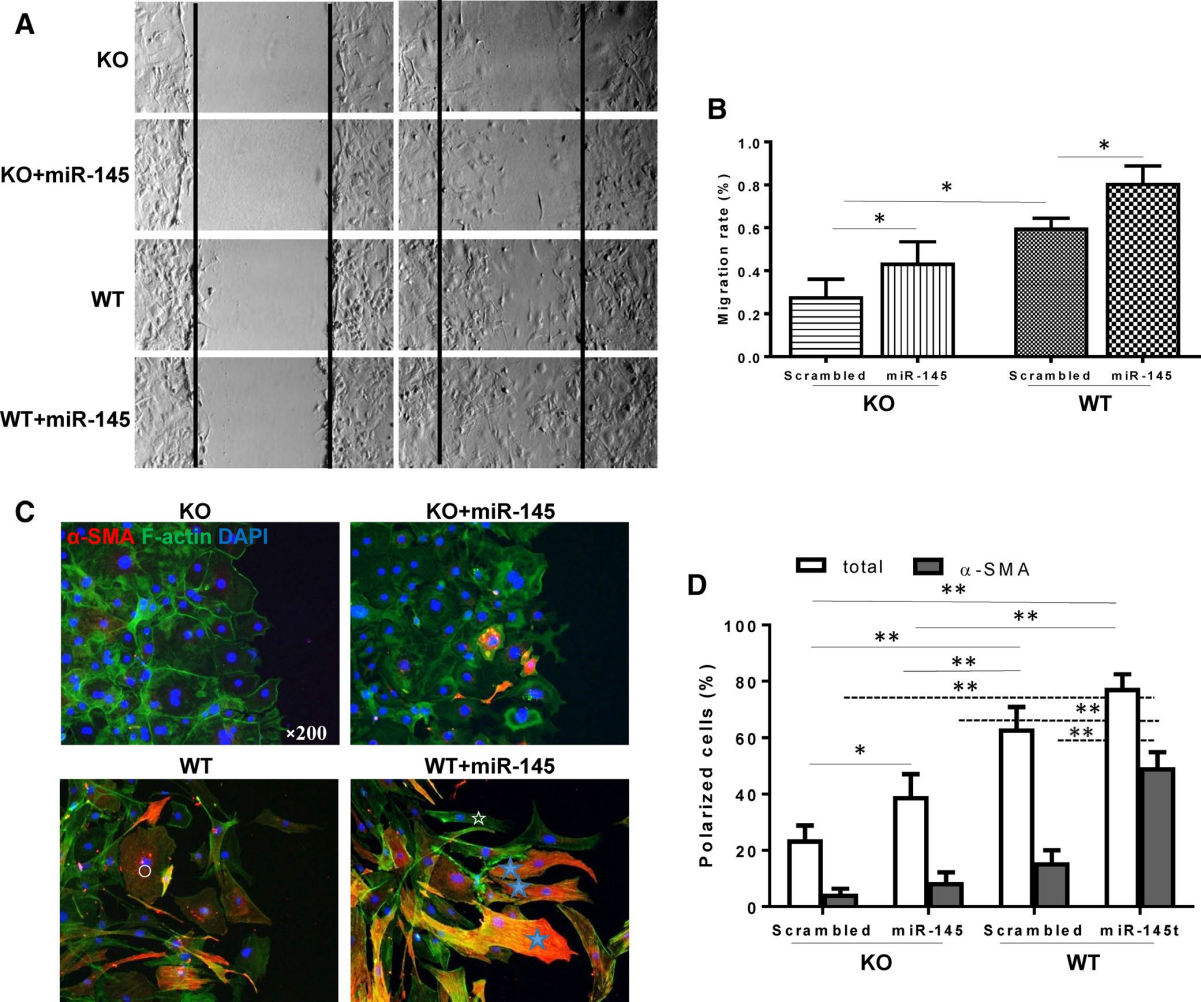
Effects of miR‐145 on the migration and polarity of cardiac fibroblasts. (A‐B) The rate of migration of cardiac fibroblasts from miR‐145 knock‐out (KO) treated with a scrambled miRNA was lower than that from wild type (WT) treated with a scrambled miRNA. Treatment with a miR‐145 mimic partially restored the migratory ability of KO cardiac fibroblasts. (C‐D) The number of polarized fibroblasts around the wound edge in KO treated with a scrambled miRNA was less than in WT mice treated with a scrambled miRNA. Treatment with a miR‐145 mimic partially restored the number of polarized cells in KO cardiac fibroblasts. ○non‐polarized cell, ☆α‐SMA^−^ polarized cell, 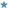
α‐SMA^+^ polarized cell, *P < .05, **P < .01, n = 5/group for B, n = 3/group for D

To detect the contractile function of the converted cardiac fibroblasts, collagen gel contraction assay was performed. Cardiac fibroblast‐mediated collagen gel contraction in KO treated with a scrambled miRNA was significantly less than in WT fibroblasts treated with a scrambled miRNA. Treatment with a miR‐145 mimic increased the gel contraction for both WT and KO compared to their respective non‐treated group, indicating increased contractile ability in both groups of cardiac fibroblasts (Figure 4C). Moreover, miR‐145 mimic treatment partially restored the ability of KO fibroblasts to contract the collagen gel (Figure 4C). Cell migration (wound healing) is another way to assess the conversion of proliferative fibroblasts to functionally active myofibroblasts (Figure 5A). The migration (by a wound‐scratch assay) of cardiac fibroblasts was lower in KO compared with WT mice, and treatment with a miR‐145 mimic partially restored the migratory ability of cardiac fibroblasts in KO and also promote the migratory ability of cardiac fibroblasts in WT mice (Figure 5B). Cells were allowed to migrate for 24 hours and then were fixed to quantify the number of polarized cells, which were identified as those with well‐oriented F‐actin‐filament bundles or α‐SMA‐filament bundles (or both) that were oriented perpendicularly within the 120° sector facing the edge of the wound (Figure 5C). The number of polarized fibroblasts around the wound edge in KO was less than that from WT mice. Treatment with a miR‐145 mimic significantly increased the number of polarized cells in the KO compared to its scrambled‐treated cardiac fibroblasts (Figure 5D). Furthermore, miR‐145 mimic treatment increased the number of α‐SMA^+^ polarized cells with the WT + miR‐145 group had the highest number of α‐SMA^+^ polarized cells. Taken together, these results implied that miR‐145 promoted the contractile function of cardiac fibroblasts, which is an important feature for the infarct scar contraction and perhaps the prevention of adverse cardiac remodelling after MI.

### 3.5 Klf4 and myocardin are the targets of miR‐145 involved in the regulation of cardiac fibroblast differentiation to myofibroblasts

**FIGURE 6:**
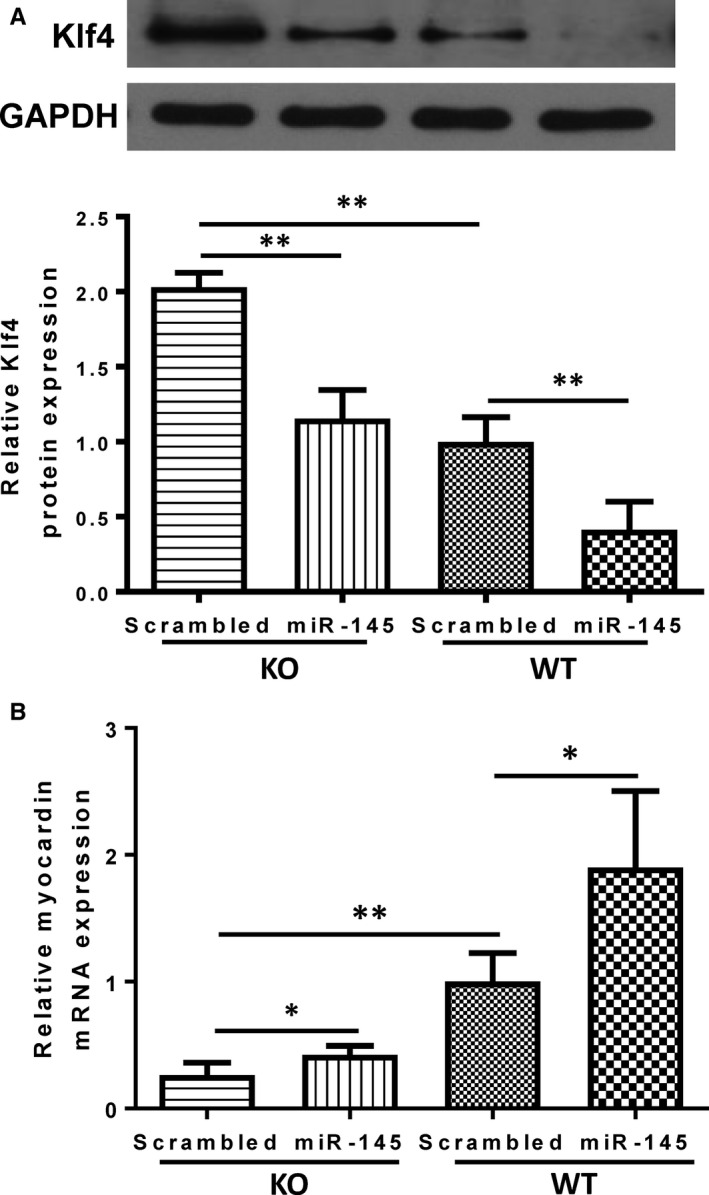
Klf4 and myocardin are the targets of miR‐145 involved in the regulation of cardiac fibroblast differentiation to myofibroblasts. (A) The protein levels of Klf4 (the target gene of miR‐145) in miR‐145 knock‐out (KO) and wild‐ type (WT) cardiac fibroblasts were examined by Western Blotting. Klf4 expression level was significantly higher in KO cardiac fibroblasts treated with a scrambled miRNA than in WT treated with a scrambled miRNA. Treatment with a miR‐145 mimic decreased the expression of Klf4 in both KO and WT cardiac fibroblasts compared to their respective controls. (B) Myocardin (the downstream mediator of Klf4) mRNA expression was evaluated by real‐time qPCR and normalized to that of GAPDH. Myocardin expression level was significantly lower in KO cardiac fibroblasts treated with a scrambled miRNA than in WT treated with a scrambled miRNA. Treatment with a miR‐145 mimic partially restored the expression of myocardin in KO cardiac fibroblasts. *P < .05, **P < .01, n = 3/group for A, n = 6/group for B

Since myocardin and Klf4 are regulators for SMC‐specific contractile protein expression,^26^ we postulated that KO of miR‐145 may inhibit the transdifferentiation of cardiac fibroblasts into contractile myofibroblasts through the same target genes Klf4 and myocardin as in SMCs. As expected, Klf4 protein expression level was significantly higher in cardiac fibroblasts of KO mice than those in WT mice, implying releasing the inhibition on Klf4 after knock‐out miR‐145 (Figure 6A). Treatment with a miR‐145 mimic decreased the expression of Klf4 in both cardiac fibroblasts of KO and WT mice with the Klf4 level in the miR‐145‐treated KO fibroblasts comparable to that of the WT (Figure 6A). Myocardin mRNA expression was evaluated by real‐time qPCR and the results showed that myocardin mRNA expression in KO was significantly lower than in WT cardiac fibroblasts. Treatment with a miR‐145 mimic not completely yet partially restored the expression of myocardin in KO compared to in WT cardiac fibroblasts (Figure 6B). These results demonstrated that KO of miR‐145 released the inhibition on Klf4 and subsequently decreased myocardin expression. On the other hand, restoring miR‐145 level in KO fibroblasts with a mimic decreased Klf4 and in turn increased myocardin expression which eventually promoted the transdifferentiation of cardiac fibroblasts into more contractile myofibroblasts.

## 4 DISCUSSION

Accumulating evidence indicates that preventing infarct scar thinning and dilatation may prevent cardiac remodelling and reduce left ventricular contractile dysfunction and improve the prognosis after MI.^5^, ^7^, ^8^ However, clinically relevant treatments to prevent heart failure are limited. Novel strategies which complement existing therapy are needed to stem the increasing number of patients with heart failure. In this study, we identified miR‐145 as a novel target for preventing ventricular dilation after MI. We demonstrated that miR‐145 was decreased in both the scar and border regions 3 days post‐MI in WT mice. We further demonstrated that cardiac function decreased more and the infarct size was larger but thinner in miR‐145 KO than that in WT mice. The increased scar thinning and dilatation in miR‐145 KO mice were associated with the thickening of the LV remote region, owing to compensatory cardiac hypertrophy. These results suggested that miR‐145 may participate in cardiac repair after MI. Previous studies have shown that miR‐145 represses the proliferation and promotes the differentiation of multiple cell types, including cancer cells, stem cells, endothelial cells and SMC etc.^20^, ^26^, ^27^, ^28^, ^29^ More importantly, studies have demonstrated the regulation of SMC status between synthetic or proliferative and contractile phenomenon by miR‐145.^26^, ^29^, ^30^ The temporal kinetic change of miR‐145 observed in the present study may reflect the dynamic role of miR‐145 involved in the progression of cardiac remodelling after MI.

We found that at the 1st stage within 3 days after MI, the level of miR‐145 decreased significantly which may result in the release of inhibition on cell proliferation. Therefore, enough cells proliferate to participate in the process of cardiac repair. This proliferation may be associated with scar maturation to retain the integrity of the ventricular wall and prevent ventricular rupture. Then in the 2nd stage from 7 to 14 days after MI, miR‐145 increased gradually until normal levels are restored. With the increase in miR‐145, cell proliferation was inhibited and differentiation was initiated. Thus, the cells proliferated during the 1st stage such as fibroblasts then trans‐differentiated to the functional contractile myofibroblasts. The final stage of MI involves the remodelling and repair of the infarct area with myofibroblast contraction and the formation of the cross‐linked mature collagen scar. All of these findings point to the important role of miR‐145 as a mediator to facilitate the transdifferentiation of fibroblasts to myofibroblasts.

Most of the cardiomyocytes of adult mammalian heart are a post‐mitotic cell types with a limited capacity for proliferation and regeneration.^31^ Cardiac fibroblasts are the most numerous cell type and interact with myocytes. In our previous study, we found that miR‐145 promotes the transdifferentiation of cardiac fibroblasts to myofibroblasts.^32^ In the current study, we used miR‐145 KO mouse model to evaluate if down‐regulation of miR‐145 was associated with decreased myofibroblast formation and increased adverse cardiac remodelling after MI. The result from immunofluorescent staining of the heart slices for α‐SMA showed that myofibroblasts in the whole heart were lower in KO mice, indicating that transdifferentiation of cardiac fibroblasts into myofibroblasts was limited in miR‐145 KO mice. When the functional capacity of cardiac fibroblasts was evaluated in vitro, the results showed that the migratory and contractile functions of cardiac fibroblasts from miR‐145 KO mice were impaired and miR‐145 mimic partially restored these functions. These results further confirmed that miR‐145 promotes the differentiation of cardiac fibroblasts into myofibroblasts, thereby preserving the ventricular scar and preventing ventricular dilatation and dysfunction after an MI.

Cell migration is accompanied by cytoskeletal polarization, and cellular polarization is indicative of increased motility. Bentzinger et al^33^ suggested that Wnt7a/Fzd7 signalling stimulates the motility of satellite cells and myogenic progenitors by inducing polarization and enhancing the directionality of migration. The promotion of cell migration and polarity is important for tissue repair.^34^, ^35^, ^36^ In this study, we demonstrated that miR‐145 promoted the migration and polarity of cardiac fibroblasts which is an important step for the process of cardiac repair.

To elucidate the mechanisms associated with miR‐145 mediated transdifferentiation of cardiac fibroblasts into myofibroblasts, we evaluated its predicted targets Klf4 and myocardin. Our study showed that Klf4 expression was significantly higher in cardiac fibroblasts of KO than in WT mice. Treatment with a miR‐145 mimic decreased the expression of Klf4 in KO cardiac fibroblasts to a level comparable to that of the WT. On the other hand, myocardin expression in cardiac fibroblasts was significantly lower in KO than in WT mice. Treatment with a miR‐145 mimic partially restored the expression of myocardin in KO mice. This finding is consistent with other studies showing that Klf4 is an important factor to reprogram human fibroblasts into a more pluripotent state,^37^ and myocardin and Klf4 are major regulators of contractile protein expression.^26^, ^29^ However, future experiments to examine the effects of miR‐145 on its targets in the PDGF and TGFβ pathways, as well as how these pathways might influence myofibroblast differentiation, are required.

## 5 CONCLUSIONS

In summary, using a miR‐145 KO mouse model, we found that miR‐145 contributes to infarct scar stabilization in WT mice and the absence of miR‐145 results in greater infarct scar thinning and dilatation, accompanied by compensatory cardiac hypertrophy. The deterioration of cardiac function in miR‐145 KO mice was accompanied with decreased differentiation of cardiac fibroblasts to myofibroblasts. In vitro evaluation using miR‐145 KO cardiac fibroblasts showed that cell polarization and migration of KO cardiac fibroblasts were lower than that of the WT mice. In addition, collagen I and α‐SMA protein levels as well as collagen gel contraction were lower in KO than WT mice. In vitro restoration of miR‐145 induced more differentiation of fibroblasts to myofibroblasts and this effect involved the target genes Klf4 and myocardin. Augmentation of miR‐145 may be an attractive target to prevent adverse cardiac remodelling after MI.

## CONFLICT OF INTEREST

The authors confirm that there are no conflicts of interest.

## AUTHOR CONTRIBUTION

**Huifang Song:** Data curation (equal); Formal analysis (equal); Investigation (equal); Methodology (equal); Validation (equal); Visualization (equal); Writing‐original draft (equal). **Sheng He:** Data curation (supporting); Formal analysis (supporting); Investigation (supporting); Methodology (supporting); Software (supporting); Visualization (supporting). **Shu‐Hong Li:** Data curation (supporting); Formal analysis (supporting); Investigation (supporting); Methodology (supporting); Writing‐review & editing (supporting). **Jun Wu:** Data curation (supporting); Formal analysis (supporting); Investigation (supporting); Methodology (supporting); Validation (supporting); Visualization (supporting); Writing‐review & editing (supporting). **Wen‐Juan Yin:** Data curation (supporting); Formal analysis (supporting); Investigation (supporting); Methodology (supporting); Validation (supporting); Visualization (supporting). **Zhengbo Shao:** Data curation (supporting); Formal analysis (supporting); Investigation (supporting); Methodology (supporting); Validation (supporting); Visualization (supporting). **Guo‐Qing Du:** Data curation (supporting); Formal analysis (supporting); Investigation (supporting); Methodology (supporting); Validation (supporting); Visualization (supporting). **Jie Wu:** Data curation (supporting); Formal analysis (supporting); Investigation (supporting); Methodology (supporting); Validation (supporting); Visualization (supporting). **Jiao Li:** Data curation (supporting); Formal analysis (supporting); Investigation (supporting); Methodology (supporting); Validation (supporting); Visualization (supporting). **Richard D. Weisel:** Conceptualization (supporting); Project administration (supporting); Writing‐review & editing (supporting). **Subodh Verma:** Resources (equal); Writing‐review & editing (supporting). **Jun Xie:** Conceptualization (equal); Funding acquisition (equal); Project administration (equal); Resources (supporting); Writing‐review & editing (equal). **Ren‐Ke Li:** Conceptualization (lead); Funding acquisition (lead); Project administration (lead); Resources (equal); Writing‐review & editing (equal).

## Supporting information

App S1

## Data Availability

The data that support the findings of this study are available on request from the corresponding author.
