# Supplementary material for: Knock‐out of MicroRNA 145 impairs cardiac fibroblast function and wound healing post‐myocardial infarction
Source: J Cell Mol Med. 2020 Jul 6;24(16):9409–19. doi: 10.1111/jcmm.15597 (PMC7417705; doi:10.1111/jcmm.15597)
Supplement: Supplementary file 1 — App S1 [file JCMM-24-9409-s001.docx]

**Supporting Information**

**for**

**Knock-out of MicroRNA 145 Impairs Cardiac Fibroblast Function and Wound Healing Post Myocardial Infarction**

Hui-Fang Song^1,2,3^, Sheng He^2,3^, Shu-Hong Li^3^, Jun Wu^3^, Wenjuan Yin^2,3^,Zhengbo Shao^3^, Guo-qing Du^3^, Jie Wu^3^, Jiao Li^3^, Richard D. Weisel^3,4^, Subodh Verma^5^, Jun Xie^2*^, Ren-Ke Li^3,4*^

^1^ Department of Anatomy, Shanxi Medical University, Taiyuan, China

^2^Department of Biochemistry and Molecular Biology, Shanxi Key Laboratory of Birth Defect and Cell Regeneration, Shanxi Medical University, Taiyuan, China

^3^Toronto General Research Institute, University Health Network, Toronto, ON, Canada

^4^Division of Cardiac Surgery, Department of Surgery, University of Toronto, Toronto, ON, Canada

^5^Division of Cardiac Surgery, Li Ka Shing Knowledge Institute of St Michael's Hospital, Department of Surgery, University of Toronto, Toronto, ON, Canada

**
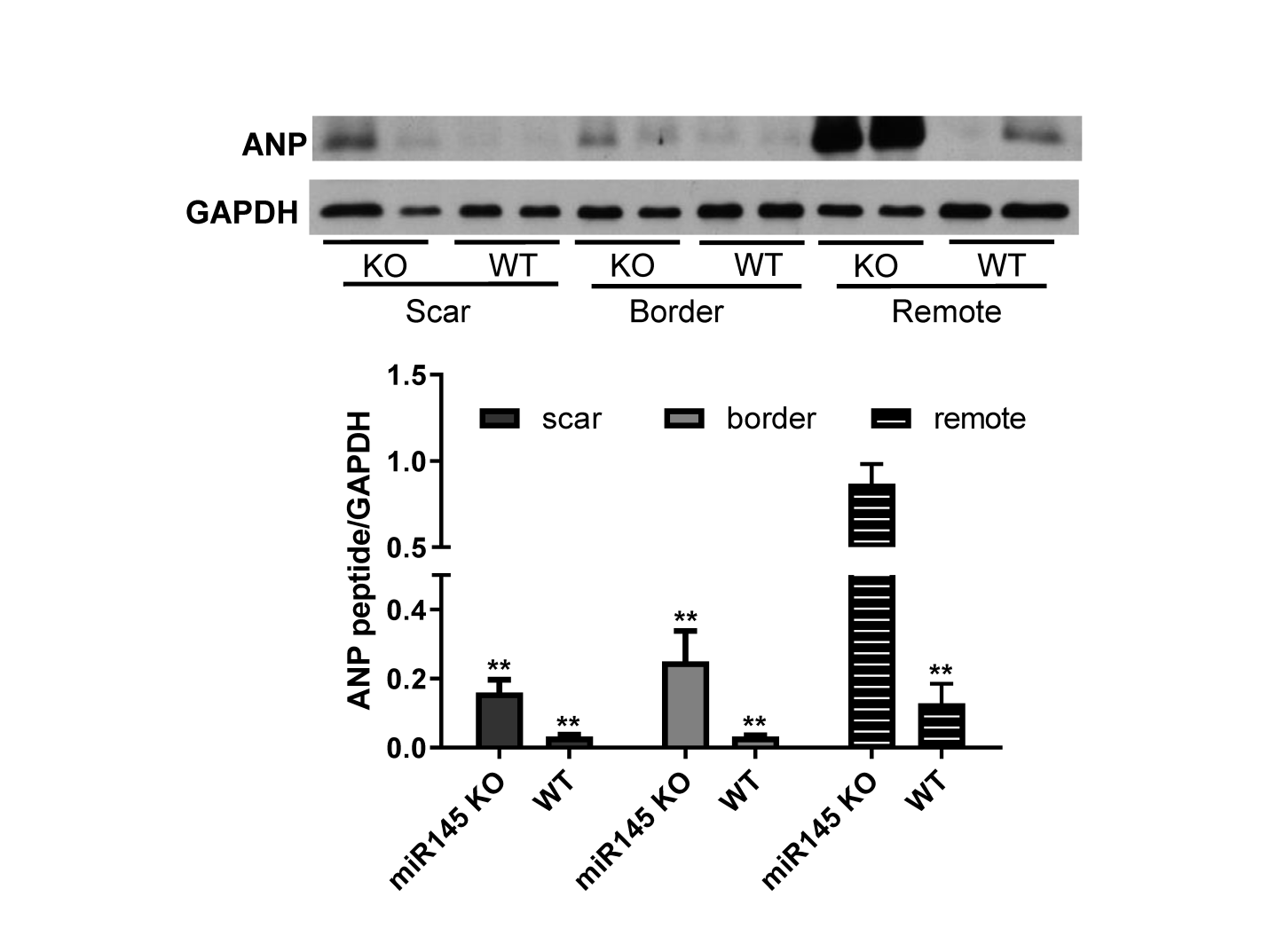
**

**Supplementary Figure 1: ANP levels in WT and miR-145 KO mouse hearts.**

Myocardial infarction (MI) was induced in wild type (WT) and miR-145 knock-out (KO) mice and the levels of atrial natriuretic peptide (ANP) was evaluated by Western Blotting at 3 days post-MI. ANP expression level was significantly higher in the remote region of the KO mouse hearts. **p<0.01 vs. miR-145 KO remote regions, n=6/group for WT, n=7/group for KO mice.
